# Detection of metallic cobalt and chromium liver deposition following failed hip replacement using T2* and R2 magnetic resonance

**DOI:** 10.1186/s12968-016-0248-z

**Published:** 2016-05-06

**Authors:** Amna Abdel-Gadir, Reshid Berber, John B. Porter, Paul D. Quinn, Deepak Suri, Peter Kellman, Alister J. Hart, James C. Moon, Charlotte Manisty, John A. Skinner

**Affiliations:** Institute of Cardiovascular Science, University College London, London, UK; Barts Heart Centre, St. Bartholomew’s Hospital, London, UK; Royal National Orthopedic Hospital, Stanmore, UK; Department of Hematology, University College London, London, UK; Diamond Light Source, Harwell Science and Innovation Campus, Didcot, UK; Department of Gastroenterology, The Whittington Hospital, London, UK; Medical Signal and Image Processing Program, National Heart, Lung, and Blood Institute, Bethesda, MD USA

**Keywords:** MRI, T2*, Metal-on-metal hip, Cobalt, Chromium, Metal loading

## Abstract

**Background:**

Failed hip prostheses can cause elevated circulating cobalt and chromium levels, with rare reports of fatal systemic organ deposition, including cobalt cardiomyopathy. Although blood cobalt and chromium levels are easily measured, organ deposition is difficult to detect without invasive biopsy. The T2* magnetic resonance (MR) method is used to quantify tissue iron deposition, and plays an important role in the management of iron-loading conditions. Cobalt and chromium, like iron, also affect magnetism and are proposed MR contrast agents.

**Case Presentation:**

We describe a case of a 44-year-old male with a failed hip implant and very elevated blood cobalt and chromium levels. Despite normal cardiac MR findings, liver T2* and R2 values were abnormal, triggering tissue biopsy. Liver tissue analysis, including X-ray fluorescence, demonstrated heavy elemental cobalt and chromium deposition in macrophages, and no detectable iron.

**Conclusions:**

Our case demonstrates T2* and R2 quantification of liver metal deposition in a patient with a failed hip implant. Further work is needed to investigate the role of T2* and R2 MR in the detection of metal deposition from metal on metal hip prostheses.

## Background

Hip replacement surgery is highly successful and 300,000 procedures are performed per year in the US alone. Sometimes they fail early. Recently a new mechanism and consequence of failure has been identified: the release of metal debris. This is common in patients with metal-on-metal (MoM) hip implants, but may occur in other implant types when ceramic/plastic components fracture.

The metal alloys used in hip implants are safe when the bulk material is considered. However, wear and corrosion of these alloys may result in the generation of metal nanoparticles and metal ions with the most clinically relevant metal elements being cobalt (Co) and chromium (Cr) in both physical forms. Metal ion release can cause local soft tissue reactions and systemic toxicity, with reports of fatal cobalt cardiomyopathy, thyroid, and neuro-ocular toxicity [1, 2]. Although the causative mechanisms remain unclear, component design, positioning, female gender, and the hypoxia-inducible factor pathway are linked to complications [3–6].

Serial measurement of serum metal ion levels in symptomatic patients with MoM hips is currently recommended by the medical device regulatory bodies (including the FDA and MHRA), and guides management including revision [2, 7]. A threshold of 7 mcg/L (118 nmol/L cobalt or 134.5 nmol/L chromium) tracks risk of local soft tissue reactions and risk of failed resurfacings/total hip arthroplasties, but there are currently no non-invasive tests for the detection of systemic or organ - specific toxicity. Confirmation of systemic toxicity has previously been from invasive tissue biopsy or post-mortem studies.

Magnetic resonance (MR) imaging using the T2* or R2 technique is the current gold-standard method for detection and quantification of iron deposition in patients with iron overload, and has histological validation [8, 9]. In such patients, the liver is the dominant site of iron storage and liver iron levels reflect total body iron stores and predict outcomes including liver and heart failure [10]. Iron exhibits ferromagnetic properties and acts like a contrast agent, relaxing water hydrogen, which is detectable as an MR signal. Both cobalt and chromium have magnetic properties and have been proposed as MR contrast agents [11, 12] and should therefore be detectable in vivo using MR T2* quantification. Cobalt like iron is ferromagnetic, and chromium is diamagnetic or paramagnetic depending on the oxidation state and this has a direct effect on magnetic susceptibility. We describe a patient with a failed hip implant where MR abnormalities raised the suspicion of remote organ (liver) involvement, with subsequent confirmation of high level tissue Co and Cr deposition on biopsy.

## Case presentation

A 44-year-old man presented to our institution with hip pain, a peri-prosthetic mass and scar pigmentation. He had initially undergone primary total hip arthroplasty 35 months previously, with a ceramic on ceramic bearing for osteoarthritis. 16 months following implantation, this was revised to a metal on polyethylene bearing implant, following a ceramic acetabular liner fracture. On presentation to our institution, laboratory investigations revealed extreme blood Co and Cr levels (587.9 mcgg/L and 20.4 mcgg/L respectively), with normal inflammatory markers. Urgent re-revision surgery to a new ceramic on ceramic bearing hip implant was performed. At surgery we found a catastrophically worn metal head (made of CoCr alloy) and substantial soft tissue metallosis. His blood ion levels reduced after re-revision surgery but were sufficient to cause concern regarding potential cobalt cardiotoxicity, and he was therefore referred for cardiac assessment using cardiovascular MR (CMR). Due to the nature of the referral with concerns of metal loading, tissue characterization sequences of the liver were also acquired.

## Investigations

### Blood tests

Blood Co and Cr levels were very high and fell with revision, (Table 1). All routine hematological and biochemical blood tests were normal throughout the follow up period, including liver function, enzymes, and iron studies. He was known to be hypothyroid prior to surgery, and blood tests found him to be euthyroid on replacement therapy.

**Table 1:** Serial blood cobalt and chromium levels with concurrent myocardial and hepatic T2*

	Scan Date (month/year)	LVEF (%)	EDV (ml)	ESV (ml)	Mass (g)	Cardiac T2* (ms) (LLN 20 ms)	Liver T2* (ms) (LLN 6.3 ms)	Equivalent liver iron concentration using FerriScan R2 (mg/g/dry tissue)	Blood Co (ULN 0.9mcg/L)	Blood Cr (ULN <0.3mcg/L)
1	Apr-14	68	142	46	127	29	4.2	3.9	9976	393
2	Oct-14	66	139	47	122	24	3.3	5.6	392	200
3	Jan-15	60	143	58	129	36	3	5.3	276	192
4	Jun-15	65	145	51	113	27	2.9	5.3	151	132

Myocardial T2* values were normal. Short hepatic T2* values (normal greater than 6.2 ms) indicate the presence of metal. Despite falling blood metal ion levels, liver MR results suggested increasing tissue deposition. Ferriscan measurements demonstrate the detectable signal change and iron equivalent for an iron-loaded patient. (*LLN* lower limit of normal, *ULN* upper limit of normal)

### Magnetic resonance Imaging Protocol

CMR was performed using a 1.5 Tesla Magnetic Resonance scanner (Avanto, Siemens medical solutions, Erlangen, Germany). Cardiac volumes and function were calculated from short-axis steady state free precession (SSFP) cine images. Pre-contrast myocardial and liver T2* maps were acquired using a single breath-hold ECG-gated multi-echo technique to generate eight (heart) and twelve (liver) images (TR: 2 msec, TE 2.59- 18.2 cardiac and minimum TE 0.99 ms liver, slice thickness: 10 mm, flip angle: 20**°**, field of view read/ phase: 400 mm/75 %). In addition, a T2 (presented as reciprocal, R2) measurement (FerriScan®) was performed through the middle of the liver in the transverse plane [13]. Late gadolinium enhancement images were acquired after administering 0.1 mmol/kg of gadolinium-based contrast (gadoterate meglumine - Dotarem, Guerbet SA, Paris, France). Following the initial study, 6 weeks post-surgery, four identical but non-contrast serial scans were performed within a fourteen-month period (Table 1).

### MR results

**Fig. 1:**
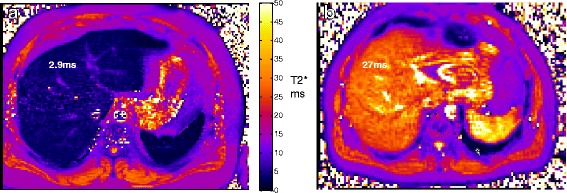
Liver T2* MR maps of patient compared with healthy volunteer. Left panel (**a**) shows the patient with a low T2* (final scan), compared to a healthy volunteer shown in the right panel (**b**) with normal T2* values

Cardiac volumetric and functional assessments were normal in all studies, with normal myocardial T2* values when compared with published values (Table 1) [14, 15]. The liver was found to have shortened signal decay by T2*, and R2 values at levels usually consistent with the equivalent of mild liver iron (Fe) overload on all scans, Fig. 1. Despite normalizing blood Co and Cr levels over the 16 month period post revision, the first three T2* values suggested worsening liver involvement (Table 1).

### Hemochromatosis DNA testing

Restriction enzyme PCR revealed the patient to be HFE compound heterozygous for C282Y/H63D, known to cause <5 % of cases of hereditary hemochromatosis. With this mutation, iron loading is uncommon without a second co-factor such as alcohol abuse or hepatitis [16], and patients have abnormal iron biochemistry [17]. Combined review by hepatology and ferro-hematology teams concluded his abnormal liver MR results were unlikely to be caused by iron loading despite the mutation.

### Liver biopsy

**Fig. 2:**
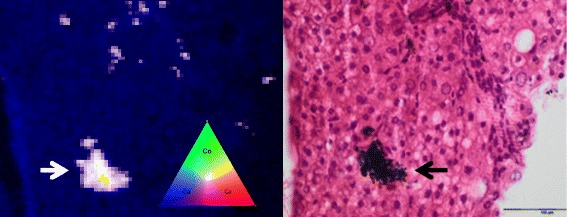
Analysis of liver biopsy tissue using micro X-ray Fluorescence (μXRF, left) found cobalt and chromium co-localised in macrophages (right). Left: μXRF element distribution color maps with 4 μm resolution - Co (*green*), Cr (*red*) and Calcium, Ca (*blue*) showing deposits (*white arrow*). The Ca distribution provides a background image of the tissue. Right: the same slide subsequently H&E stained showing aggregates of pigmented macrophages (*black arrow*)

The patient underwent ultrasound-guided liver biopsy. Care was taken to avoid metal contamination with Fe, Co or Cr. Liver tissue was analyzed at 3 separate reference laboratories for histology, iron quantification, and Co-Cr detection. Standard liver histology was within normal limits and no fibrosis, no iron or copper was identified, however apparent metal deposits were detected in liver macrophages (Fig. 2).

To determine the exact location and chemical composition (speciation) of this, we performed micro-X-ray Fluorescence (μXRF) and micro X-ray Absorption Spectroscopy (μXAS) on beamline I18 at Diamond Light Source (Harwell, UK) [18]. μXRF elemental mapping causes minimal sample damage, is sensitive to very low concentrations, and allows the samples to be later treated by conventional histology techniques for co-registration. μXAS can then provide details on the speciation. These showed an abundance of highly co-localized Co and Cr metallic particulate debris in macrophages with no isolated Cr or Co (Fig. 2). The particles were found to have uniform composition across samples, but an enhanced Co to Cr ratio (4.1:1) compared to the source hip (ASTM F75 alloy typically Co:Cr 2.25:1).

## Discussion

We present the first description of the use of T2* and R2 MR to non-invasively detect and map liver Co and Cr. Our patient had extremely elevated blood metal levels from a failed hip prosthesis with wear of the Co-Cr head and the highest values seen by their attending surgeons at a large specialist retrieval unit in London. Liver biopsy followed by μXRF and μXAS confirmed Co-Cr debris in liver macrophages, with no other ferromagnetic material deposits found. Serial assessment suggested clearance from the liver was far slower than from the blood.

More than one million people globally have MoM hip prostheses and are “at-risk” of Co-Cr release. This causes a high failure rate due to local soft tissue reactions causing pain and pseudotumors. The failure rate of these hips can be up to 40 % by 7 years [19], and implantation of these has now all but ceased. Co-Cr release may also occur with other types of prostheses, here due to ceramic fracture - although improved surgical technique, implant design and materials are making this uncommon. In addition to local complications, there is concern from clinicians and patients alike regarding potential systemic toxicity from the high blood Co and Cr. Systemic organ deposition is hard to measure without invasive biopsy, meaning that currently available evidence is not always robust. This uncertainty coupled with medico-legal influences have fuelled patient anxiety, are driving some aspects of management, and highlight the need for non-invasive tests for systemic organ deposition.

This case illustrates that systemic deposition of Co and Cr in the liver can occur in subjects with extreme blood levels of metal secondary to failed hip implants, even without blood markers of hepatic abnormality. The absence of liver enzyme disturbance suggests that this patient tolerated the Co and Cr, at least in the short term. The relationship of liver deposition/sequestration to other organ toxicity (brain, thyroid, spleen etc.) is unknown. In iron loading states the deposition of iron occurs primarily in the liver followed by deposition in other organs including the heart. A similar pattern may be seen in patients with increased levels of blood Co and Cr. The relative proportions of Co and Cr suggest progressive particle processing during the transport from the hip to the liver, although this mechanism remains unknown.

T2* and R2 MR are currently routinely used to detect and quantify iron tissue deposition in the heart and liver, and have significantly changed disease outcomes. Iron deposition with T2* MR of the pancreas, pituitary and kidneys is also potentially quantifiable. These techniques could also be repurposed for non-invasive screening of Co-Cr deposition in at-risk patients. To date, only non-specific qualitative abnormalities have been reported on CMR scans in patients with biopsy proven Co cardiomyopathy [20, 21]; the reported changes likely reflecting the myocyte response and inflammation rather than deposition. A non-invasive tool for Co/Cr organ deposition, if sensitive, would likely influence clinical decision-making. Whether the non-invasive detection as described here works only in extreme cases, or whether liver (or other organ) metal quantification may inform about risk in that or other organs is unknown. Further research is needed. A limitation of this report is that Fe, Co and Cr levels were not quantified in the liver biopsy for comparison with the MR findings.

## Conclusions

Over one million patients with MoM hip prostheses are at risk of cobalt/ chromium toxicity with potentially devastating complications. The T2* method plays an integral part in the management of patients with iron overload, and this technique may also detect CoCr deposition in the liver as demonstrated by our case. Further work is needed to assess the utility of T2* and R2 MR as a screening tool in at-risk patients with MoM hip prostheses.

### Consent

Written informed consent was obtained from the patient for publication of this case report and any accompanying images.
